# Reply to Comment on “Differential Effects of MitoVitE, α-Tocopherol and Trolox on Oxidative Stress, Mitochondrial Function and Inflammatory Signalling Pathways in Endothelial Cells Cultured under Conditions Mimicking Sepsis. *Antioxidants* 2020, *9*(3), 195”

**DOI:** 10.3390/antiox9060464

**Published:** 2020-06-01

**Authors:** Beverley E. Minter, Damon A. Lowes, Nigel R. Webster, Helen F. Galley

**Affiliations:** Institute of Medical Sciences, University of Aberdeen, Aberdeen AB41 8TJ, UK; bminter2@sky.com (B.E.M.); damon.lowes@rothamsted.ac.uk (D.A.L.); n.r.webster@abdn.ac.uk (N.R.W.)

We thank Drs Hegarty and Byrne for their interest in our paper and appreciate the opportunity to respond to their insightful comments. We have addressed each point in the order in which they were raised.

1. Hegarty and Byrne [1] state that Alamar Blue should not be used for the determination of ‘’mitochondrial function’’ when studying vitamin E, because Alamar Blue works via a redox-based mechanism. As noted, resazurin, the active compound in Alamar Blue, acts as an electron acceptor in the electron transport chain within the inner mitochondrial membrane and can be reduced to the fluorescent compound resorufin by some antioxidants, with spurious results in the presence of very high concentrations of antioxidants [2]. As part of our preliminary assay development and optimization, we undertook a series of experiments in the presence of a range of antioxidants including various forms of vitamin E, both with and without LPS/PepG. We did not find that antioxidants affected the rate of resazurin reduction at the concentrations we tested. In addition, experiments where culture media containing antioxidants was removed, the cells washed with PBS, and fresh media replaced prior to assay, showed no difference in the rate of reduction of resazurin whether antioxidants were present or not.
2. In our study [3], we used carboxy-DCFDA to measure the overall total cellular ROS production. Hegarty and Byrne suggest that we could have also used a mitochondrial-specific dye such as MitoSOX red. As they will be aware, this dye is taken into mitochondria using the TPP cation, the same as MitoVitE. We find that profound mitochondrial membrane disruption occurs when MitoSOX red is used with antioxidants coupled to TPP such as MitoVitE or Mito Q. We agree that investigation of peroxiredoxin (PRDX) 2 and 3 would have given additional information on subcellular activity of the forms of vitamin E.
3. In our paper [3], we report a single concentration of the forms of vitamin E as proof of concept, as noted by Hegarty and Byrne. Our preliminary studies included the determination of the optimum concentration for maximum effects. As part of this, we of course assessed the toxicity of the three forms of vitamin E at concentrations of 0.25–15µM. There was no loss of cell viability at 5µM for any of the forms of vitamin E, including MitoVitE. Viability data are presented in our supplementary data available online.
4. Hegarty and Byrne state that we did not undertake gene expression under control conditions, i.e., in the absence of LPS/PepG. However, the data presented in Figure 3 and Table 1 in our paper show differential expression in the presence of LPS/PepG compared to vehicle control as stated; our aim was to determine the relative effects of the three forms of vitamin E under conditions of sepsis.
5. Hegarty and Byrne are correct in that there are other biologically active forms of tocopherol; however, α-tocopherol is the most abundant in the body and therefore arguably the most important and a good choice for comparison, although we concur that investigating the relative effects of other tocopherols and tocotrienols would have been interesting. We agree that MitoVitE has been shown to be more protective than Trolox; nevertheless, it has been demonstrated that Trolox has potent antioxidant activity in several different models (references 7–9 in our paper [3]). We expected Trolox to be of less benefit than MitoVitE under conditions of sepsis in our model, but that was not what we found.
6. We agree that our gene expression data has generated a lot of questions, including regarding effects on the inflammasome and prostanoid biosynthesis. We concur that these potential mechanisms are certainly future avenues for further study.
